# Neglected Actors in Neglected Tropical Diseases Research: Historical Perspectives on Health Workers and Contemporary Buruli Ulcer Research in Ayos, Cameroon

**DOI:** 10.1371/journal.pntd.0004488

**Published:** 2016-04-21

**Authors:** Guillaume Lachenal, Joseph Owona Ntsama, Daniel Ze Bekolo, Thomas Kombang Ekodogo, John Manton

**Affiliations:** 1 SPHERE UMR 7219, Université Paris Diderot and Institut Universitaire de France, Paris, France; 2 Fondation Paul Ango Ela pour la Géopolitique en Afrique Centrale, Yaoundé, Cameroon; 3 Hôpital Régional Annexe d’Ayos, Ayos, Cameroon; 4 Hôpital Régional Annexe d’Ayos, Ayos, Cameroon; 5 Centre for History in Public Health, London School of Hygiene and Tropical Medicine, London, United Kingdom; Fondation Raoul Follereau, FRANCE

## Introduction

“We are neglected, just as Buruli ulcer is neglected” (WS1.KE). With these words, Kombang Ekodogo and Daniel Ze Bekolo, two nurses with long experience with Buruli ulcer disease (henceforth, BU) at the hospital of Ayos, Cameroon, expressed dissatisfaction about their involvement in international research projects. They felt that their contribution to crucial progress in the surgery, rehabilitative care, and epidemiology of BU was insufficiently recognized and rewarded. Although medical research projects meant considerable benefits for themselves and their communities, they insisted on their frustration with being, in the best cases, merely “acknowledged” in a footnote to the final publications. “When I tell my children that I participated in this and that project, they will ask: yes, dad, but where is your name [on the paper]?” (WS1.ZB).

Collaborative research in Africa faces difficult ethical challenges. Inequalities between local and expatriate scientists constrain the functioning of research projects and contradict their official presentation as “partnerships” [1]. In spite of efforts to acknowledge and redress such inequalities among researchers, the “collaboration equation” remains hard to balance [2]. Debates over the ethics of transnational research rarely take into account the case of auxiliary staff such as nurses, laboratory technicians, research assistants, and fieldworkers. Although largely invisible in academic outputs of medical research, they play an essential role at the interface of community work, medical care, and scientific research [3]. Their pivotal contribution to disease control, including HIV, tuberculosis (TB), onchocerciasis, dracunculiasis, and BU [4–9] is beginning to be acknowledged, as well as their crucial role as mediator of research ethics [10–14]. Their epistemological role as knowledge-producers has been less studied. In this research, we document the expertise of auxiliary health workers involved in BU research. We retrace historically how their knowledge about tropical diseases like leprosy, sleeping sickness, and BU has been transmitted across century-old genealogies of local nurses. This article aims to recognize and value this memory and expertise.

The original idea for this paper emerged from repeated conversations between our team of historians and anthropologists and nurses working at Ayos Hospital. Our discussions focused on nurses’ experiences of global health research, in the past and in the present. Since the 1960s, the area of the Nyong River basin (including the towns of Ayos and Akonolinga) has been identified as an endemic focus of BU [15]. BU is a mycobacterial infection causing severe skin wounds resulting in long-term disabilities, whose exact transmission mechanism is not yet elucidated [16]. It is restricted to confined foci sharing specific geographical and ecological characteristics. Ayos and Akonolinga have been key sites of transnational research on BU, which has boomed in Cameroon since the early 2000s, involving major global health actors and dozens of local health workers. Research conducted in the Nyong valley area has contributed significantly to progress in treatment and prevention strategies, including innovative approaches in therapy [17] and in the understanding of the biological, social, and environmental factors of BU emergence [18–24].

This article exposes the perspective of nurses and fieldworkers on their experience of international research on BU in Ayos and characterizes their contributions to scientific and clinical progress. This angle offers, in hindsight, an “alternative” history of BU research in Ayos. The scientific history of BU in Cameroon is often told as the tale of two discoveries, the first in the late 1960s and the second in the 2000s [25], separated by three decades of neglect and stagnation. In contrast, we propose the examination of long-term continuities in neglected tropical disease (NTD) care and research in Ayos over the 20th century. This paper discusses how BU has been a continuing concern for local healthcare workers, who developed an under-appreciated expertise in BU detection and care in the 1970s and 80s. This expertise is articulated to (1) a local historical memory linked to the standing of Ayos as a prominent medical site since the early 20th century and (2) to direct, intimate experiences of other NTDs as patients or care-givers, including human African trypanosomiasis (HAT) and leprosy. Memory is a crucial aspect of the nurses’ role at the research–community interface; it contrasts with (and compensates for) the structural amnesia of international research projects.

Finally, this article experiments with new ways of acknowledging the scientific contribution of “neglected” actors of NTD research: through a process of participatory writing, two retired nurses are included as co-authors of this article.

### Ayos, Cameroon: A Century-Old Site of Science and Medicine

Ayos has been a medical town since its creation. The medical history of Ayos dates back to the early 20th-century period of German colonialism, when a severe epidemic of HAT—the disease historically known as sleeping sickness—swept the whole Upper Nyong forest region [26–28]. The Nyong River was the main channel of transportation between the resource-rich forests of East Cameroon and the Atlantic coast, via Mbalmayo. Colonial rule and the violent rubber boom displaced populations, and the use of forced labour and long-distance carriage were the main factors of an extremely grave HAT epidemic in the Upper Nyong area. In 1913, the doctor-in-chief for German Kamerun, Philalethes Kuhn, chose Ayos as the centre of an ambitious program of sleeping sickness control covering the whole colony [29]. The hilly site, known as *Ajoshöhe*, enabled both immediate access to the river and isolation from tsetse flies, as well as strategic vantage on the whole region (OI.A1).

World War I interrupted the German program and aggravated the trypanosomiasis epidemic in the area [30]. From 1919, the French doctors took over the camp at Ayos and re-launched from there a sleeping sickness control programme based on a mobile medicine approach, which received considerable financial support and media coverage from Europe between 1925 and 1930, under the leadership of the French colonial doctor Dr Eugène Jamot [31]. In the 1920s and 1930s, Ayos served as a segregation camp (also known as a “*hypnoserie*”) and as a logistical base, experimental site, and training centre for the medical teams. The site required the presence of large numbers of nurses, workers, and their families, leading to the emergence of Ayos as a town. The male nurses working in the mobile teams of the *Mission Jamot* in the 1920s and 1930s drew considerable social prestige and economic opportunities from their professional status [32]. They are still remembered as the “*Jamotains*.”

**Fig 1:**
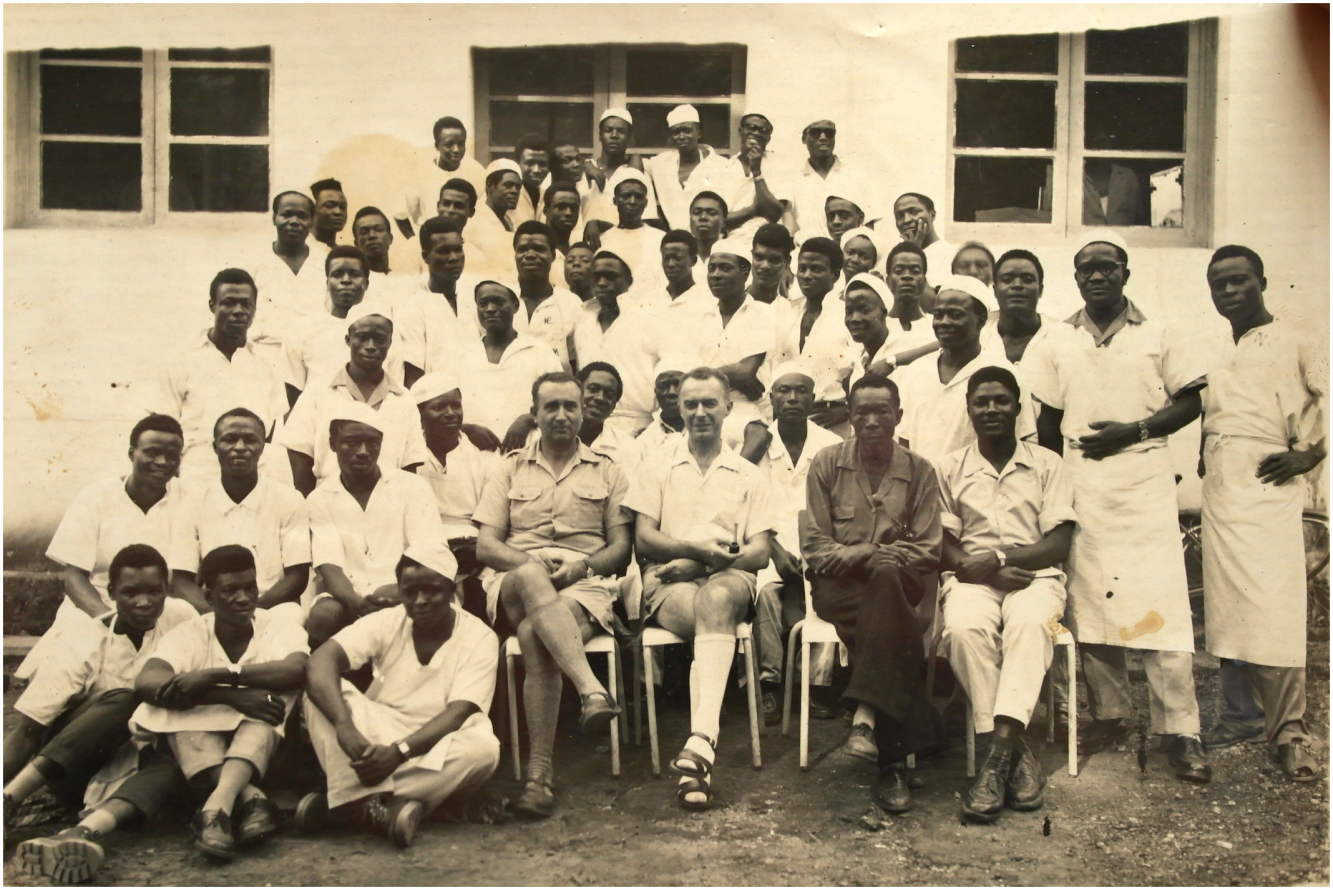
A promotion at Ayos nursing school in the 1950’s. Image used with the authorization of the Samba family.

**Fig 2:**
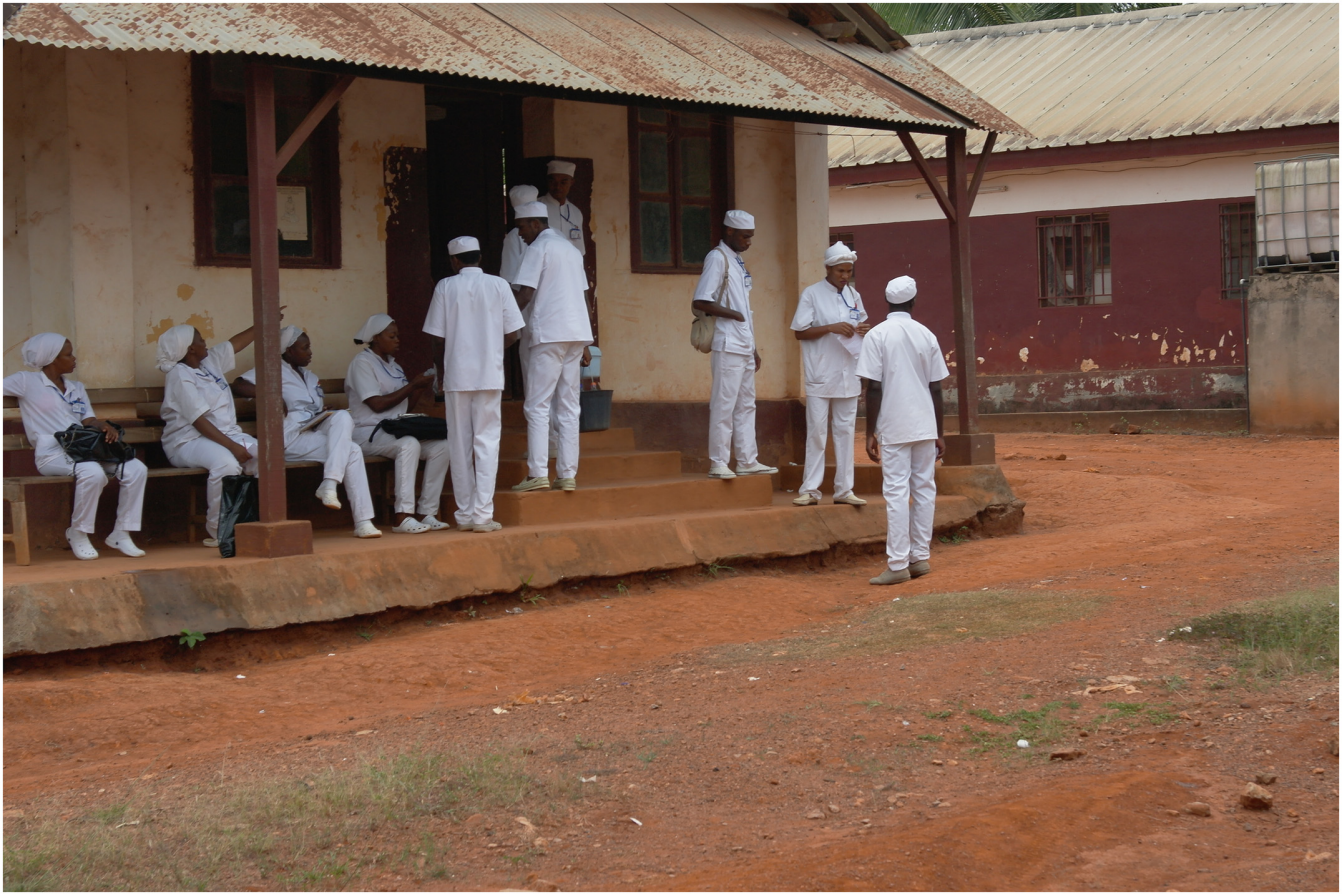
Nursing students in Ayos Hospital. Image credit: John Manton, 2013.

The need for a large medical workforce led to the creation of the *Centre d’Instruction Médicale d’Ayos* in 1932. Since then, generations of nurses have been trained in Ayos (Figs 1 and 2), including the first cohort of Cameroonian medical doctors, trained in Dakar and France in the 1950s and 1960s, and prominent Cameroonian political leaders, such as Prime Ministers Charles Assalé and Simon Pierre Tchoungui.

### Between History and Memory: Studying the Presence of the Past in Ayos

This study is part of a project bringing together historians and anthropologists studying medical research in three African sites located in Senegal, Tanzania, and Cameroon. In the three sites, the Memorials and Remains of Medical Research in Africa Project (MEREAF, 2011–2015) aims at documenting how scientists, staff, and populations relate with the past of their local institution. The site of Ayos was chosen because of its significance as a major colonial hospital and nursing school since the 1920s. How is this past present in the contemporary landscape? How does it shape contemporary practices and experiences of medical research?

Our project combined ethnographic fieldwork, interviews, and historical research. Our Cameroon team was composed of three researchers specialized in the history and anthropology of medicine: John Manton (JM), Guillaume Lachenal (GL), and Joseph Owona Ntsama (JON), assisted by Valentin Angoni (VA), logistician and translator. The full team conducted two 15-days stays in Ayos in March 2012 and March 2013, complemented by supplementary visits by JON in 2012, 2013, 2014, and 2015 and by GL in 2013 and 2015. During our visits, Daniel Ze Bekolo (ZB), retired nurse and former Leprosy and Tuberculosis Control Officer for the Nyong-et-Mfoumou Department, guided our work in the town of Ayos, and Kombang Ekodogo (KE), Nurse in chief (*Surveillant Général par intérim*) of the Annex Regional Hospital of Ayos, facilitated our research within the hospital premises.

We combined several qualitative methods: 16 in-depth interviews with active and retired hospital staff, nursing students, and the local families of former nurses, as well as informal discussions and participant observation. After oral consent, interviews (1–2 hours long) were conducted in French (with occasional translation from Ewondo and Yebekolo) and focused on the life histories of interviewees, on their knowledge of the history of medicine and nursing in Ayos, and on their experience of international research. Following a “microhistorical” approach, we considered their biographies as “ordinary exceptions,” both outstanding and revelatory of general processes [33,34].

Archival and bibliographic research was conducted in France and Cameroon to document the history of the Ayos Hospital. Relevant sources were collected at the Centre des Archives d’Outre Mer (Aix en Provence, France), Institut de Médecine Tropicale du Service de Santé des Armées (Marseille, France), the National Archives of Cameroon (Yaoundé), the Service de Coopération et d’Action Culturelle (Yaoundé), and the Centre Pasteur du Cameroun (Yaoundé). Three interviews with retired expatriate doctors were conducted in France.

### Experimenting with Participatory Writing: The Ethics of Co-authorship

Our research not only aimed at retracing the history of Buruli ulcer research in Ayos but also experimented with a process of participatory writing to document and value the scientific contribution of auxiliary health workers.

Oral interviews (OI) were transcribed into French and analysed thematically. They are quoted anonymously (e.g., OI.A1) or not (e.g., OI.ZB) according to the recorded wishes of participants. Transcripts and recordings of the interviews as well as photographs taken during interviews and visits were made available to participants during our second visit to the site. The preliminary report of our research was presented orally and discussed on two public occasions with the hospital staff and authorities, and with the interested inhabitants of Ayos, on March 9th and 12th, 2013.

Preliminary analysis identified participation and authorship in BU research as a key issue. We discussed informally with KE and ZB the possibility of a jointly authored paper on this theme. A three-hour workshop was organized in Ayos on March 10th, 2013 with the two nurses to discuss the angle, content, and outline of the present paper. This workshop was recorded (quoted as WS1). The title of the paper was suggested spontaneously by KE. A first draft was written in 2014 by GL and JM, back-translated into French, and discussed with JON, VA, and the local Ayos authors, ZB and KE, during a second workshop (WS2) in December 2013. The final manuscript was discussed in Ayos with the same, on May 24th, 2015.

Our historical research did not include present patients and did not interfere with the clinical activities of the hospital. For the purposes of this paper, permission to identify and cite Kombang Ekodogo and Daniel Ze Bekolo by name has been granted in accordance with authorship and ongoing review of draft texts. The interviewers sought oral consent from all interviewees, in presence of the full team and at least one witness. We recorded agreement, as part of the fieldwork process, in our notes. Oral consent was preferred because of the historical nature of our work: the interviewees were not put at any risk of being harmed in their safety or psychological wellbeing, and the act of signing one’s name before providing personal, economic, and occupational data could lead to mistrust and misconceptions about the nature of the research.

The MEREAF project was approved by the National Ethics Committee of Cameroon (027/CNE/SE/2012) and the Observational/Interventions Research Ethics Committee at the London School of Hygiene and Tropical Medicine (LSHTM Ethics Ref: 6067). Both committees approved the full MEREAF protocol, which referred explicitly to the ASA (UK Association of Social Anthropologists) guidelines statement about consent in ethnographic research. Since the ASA guidelines do not distinguish oral and written forms of consent (oral consent being the normal practice in ethnographic work), the approval of the former was implicit. Following the ASA statement, we consider that “consent in ethnographic research is a process, not a one-off event, due to its long-term and open-ended qualities” [35], and we sought repeated interactions with participants over a three-year period, including three public presentations of our results in Ayos to all research participants. All 17 participants gave written approval for the use of archival and photographic material in the publication of the project.

### Histories of BU Research in Ayos: Our Findings

#### 1. An alternative trajectory of BU research in Cameroon

Interviews highlighted the social context of the discovery (and rediscovery) of BU in Ayos. The first identifications of BU in Cameroon occurred in the post-independence period, at a time when scientific collaborations inherited from colonial times connected Ayos with Yaoundé, 200 km from Ayos, and Paris. BU was first reported in 1970 in the *Annual Report of the Pasteur Institute of Cameroon* (IPC) (p.51, [36]), with cases “curiously limited to the region of Ayos” (p. 49, [37]). In 1969, the director of the Ayos Hospital, an expatriate military doctor, Dr Lebourthe, referred cases of atypical ulcers to Yaoundé, where the French experts cultivated and identified *Mycobacterium ulcerans* (OI.P1) [38,39].

There was no “eclipse” of BU in Ayos between 1970 and 2001. BU surgery was practiced in Ayos from the late 1970s. The development of surgical techniques, involving extensive skin grafting, is associated in oral accounts with Dr Titus Edzoa, who worked in Ayos from 1978 to 1982 (OI.ZB) before becoming a famous political figure in Cameroon in the 1990s. In their oral accounts, nurses explained that Prof. Edzoa taught them the techniques of anaesthesia, surgery, and grafting linked to the surgery of BU (then referred to as “mycobacteria ulcer” (*Ulcère à mycobactéries*, UAM). “Cut wider! Don’t be afraid!” was the advice Edzoa would give to nurses, to explain to them the need for aggressive and extended skin removal around the BU lesion (OI.ZB). Edzoa appears as the founding figure of a genealogy of BU surgeons in Ayos (all of them nurses by professional status).

From 1986, the presence of BU was reported repeatedly by the local Leprosy and Tuberculosis District Officer, Daniel Ze Bekolo, and by his colleague Simon Bitoto. As part of their surveillance work, they conducted regular rounds to screen for cases of leprosy and tuberculosis and to monitor the treatment of existing cases. They had an intimate knowledge of the area and of the local population. They were able to identify UAM and link it to the infection known locally as *Atom*, for they had treated the disease surgically at the Ayos Hospital. As Daniel Ze Bekolo remembers:

“We would fill a form, every trimester, after it was every semester, and then monthly. There was a line where we were asked to signal if there was another noticeable infection in the area… It is on this line that I was able to signal constantly the presence of UAM at the time”

(OI.ZB).

“We signalled, we signalled, we signalled”

(OI.ZB2).

In 1998, the Regional Supervisor for Leprosy and Tuberculosis Control (*Superviseur Provincial)*, Jacques Kenne, who had regularly transmitted their reports upward to the health authorities, told Ze Bekolo and Bitoto that “their call had been heard” (OI.ZB), alluding to the launch of the Global Buruli Ulcer Initiative by WHO. Two years later, in 2000, he asked them to join the international study on Buruli ulcer in the Nyong area, involving the Cameroonian Ministry of Health, the Centre Pasteur du Cameroun (formerly known as IPC), the Swiss Tropical Institute in Basel, the Institute of Tropical Medicine in Antwerp, and Medecins sans Frontières, which marked the beginning of the renewed interest in BU in Cameroon:

“[Jacques Kenne] came and told me: ‘is there really Buruli ulcer here in Akonolinga?’ Well, I told him, ‘I don’t know this disease, Buruli ulcer, I have never heard of it.’ At this moment he understood and said ‘no, no, I know these are strange words to you, I am talking about Mycobacteria ulcer, is there any of it?’ I told him, ‘Oh yes, there is, I even have some cases currently.’ He asked me: ‘Can you find some cases? We are coming back next Thursday, do your best.’ I only searched a little in town and found six cases. [The researchers] came back, they took samples and brought them to Basel, if I remember well. All six cases were positive. This is when the machine started”

(OI.ZB).

#### 2. Clinical knowledge and diplomatic know-how: the contribution of health workers to research

Ze Bekolo and Simon Bitoto played a major role in the first epidemiological survey of BU in the Ayos-Akonolinga area (published in 2004), which identified more than 400 cases [25], since they knew personally a great number of patients with active or healed BU. In other words, the rapid “rediscovery” of BU owed to their clinical competences in diagnosis, acquired since the late 1970s, and to their personal knowledge of the space, ecology, language, politics, and culture of the Ayos-Akonolinga area.

The competencies of local health workers, including Ze Bekolo and Kombang Ekodogo, a nurse trained in BU surgery in the 1980s in Ayos, were crucial to most BU research and intervention projects that took place during the “Buruli boom” of the last decade. They were acknowledged as such in the incidental metadata (e.g., lists of acknowledgments) of certain publications concerning Ayos [15,25].

Their key skills were very diverse, ranging from technical mastery of surgical techniques to the linguistic and social skills necessary to access villages and homes, to interact with traditional healers, authorities, and patients, and to facilitate the work and daily life of expatriate researchers. They were all the more important because most research necessitated active case-finding in villages of the area, built on relations of trust with local health workers. Their clinical competences made them critical authors, rather than mere executants, of clinical guidelines and research questions. For example, they had noticed early the severity of BU in patients living with HIV, and regularly asked for research to be done on this now well-documented point (WS2.KE) [40]. The experimentation with thermotherapy (treatment of wounds by heat application using systems similar to commercial pocket heat pads) [17] was indebted to nurses’ minute adaptation of devices and bandages to lesions. Their long-term experience in attending BU wounds considerably accelerated the trial and error process, leading, for example, to the replacement of aluminium foil (as was initially planned) with cotton cloths (WS2.KE).

Health workers such as Kombang, Bitoto, and Ze Bekolo became well-known in the area, earning the respect and gratitude of former patients. Reciprocally, they had a personal memory of many cases: when we tried to read together scientific publications from Ayos BU studies [17,25], they were able to tell the story of individual patients from pictures of single lesions, which shows their key role in interfacing technical interventions and human experiences of disease and healing: “I know this hand! He partied so much after he left the hospital!”; “whose foot was it?” (WS2.ZB). They literally knew lesions by their names.

Although their involvement in research was described by nurses as largely positive, enabling them, for example, to travel within Cameroon as part of national surveys and control programs and, on one occasion, to Switzerland (OI.KE), it also exposed them to accusations. As many scholars have documented, BU is often interpreted as a mystical disease in the area [23]: *Atom*, the name of BU in local *Beti* languages, refers directly to the fact that the disease has been “sent” as part of a witchcraft attack. In this context, health workers taking part in the detection, referral, and treatment of cases are “logically” accused of being themselves at the origin of the disease [11,41,42].

“I have been called a witch, because they cannot understand that someone accepts to take care of this disease”; “[they say] that the one who works in mystical diseases must be mystical too”

(WS1.KE).

“People say that [case-finding] surveys are occasions for us to send the disease to people. This is our daily experience! According to them we were getting rich! Sending BU was a way to make money for us!”

(WS1.ZB).

This uncomfortable position requires considerable negotiation and explanation, especially when studies involve blood sampling, which is often viewed suspiciously in African communities. ZB remembers having to “teach by example and have his blood sampled twice a day so that people trust the team.” Such examples further underline the difficulties of “dual accountability” [3] and the importance of their diplomatic skills in the daily running of BU research and care in Ayos.

Nurses play a significant role as knowledge producers. The labour of nurses, as assessors of the likely success of technical and therapeutic interventions, as facilitators of intervention and epidemiological practice, and as technical innovators and “improvisers” in resource-poor settings, has a clear epistemological value, which enables, complements, and enriches the conduct and reception of scientific research per se. This contribution is recognized both by research teams, who are clearly aware of the precious value of staff such as KE and ZB, and by populations, who would, for example, address them as “docta” (from the pidgin English for doctors, widely used in French-speaking Cameroon)—but the way this recognition is expressed remains entirely local. The nurses’ absence from the authors’ list of final research reports does not result from a deliberate attempt to suppress them, but rather from the automatic reiteration of a tacit norm of international collaborative research, a norm that is thereby systematic; whatever its intent, it operates and is experienced as an exclusionary mechanism. Although it bears very little on their local social and professional status, this silence does constitute, from the nurses’ perspective, a form of neglect, that operates at the “global/local” interface.

#### 3. The making of an intimate expertise: biographies of BU health workers

The acquisition of the knowledge and know-how described above can best be understood when situated in the life-histories of the health workers co-authoring this paper. (Tables 1 and 2).

**Table 1:** Biography of Daniel Ze Bekolo.

25 January 1951	Birth in Ayos Hospital Maternity.
1958–1968	Schooling in Ayos and Mbalmayo.
1966	Experience of BU lesions as a schoolboy. Treated at Mbalmayo and Ayos Hospital.
1972–1973	Secondary schooling in Yaoundé, Ndi Samba College.
1974	Admitted to Ayos Nursing School.
1976–1978	Nurse (*infirmier breveté)*. Appointed in Evodoula rural health centre, Lekié Department.
1978–1986	Appointed as head nurse (*infirmier major*) of Operating Theatre, Ayos Hospital. Learned and practiced BU surgery (including anaesthesia and skin grafting). Trained students, including Simon Bitoto.
1985	Began working at Ayos leprosy camp.
1989	Successful surgery of his son’s BU lesion, identified as UAM.
January 1986	Training on leprosy and tuberculosis at the Organisation de Coordination et de Lutte contre les Endémies en Afrique Centrale (OCEAC), Yaoundé.
1986–2006	Leprosy and Tuberculosis District Officer for Akonolinga Health District and Nyong-and-Mfoumou Department based in Akonolinga District Hospital (supervises more than 300 cases). His colleague Simon Bitoto was in charge of Ayos leprosy camp and served as Leprosy and Tuberculosis District Officier for Ayos Health District.
2001–2002	Involvement in the first epidemiological studies of BU in Ayos.
2006	Retirement from civil service.
2007	Appointed as community health worker for BU at Aide aux Lepreux Emmaus Suisse (ALES; later Fairmed).

**Table 2:** Biography of Kombang Ekodogo.

07 November 1962	Birth in Ayos leprosy camp. His parents were leper patients who arrived at the camp in their youth (in the 1920–1930s).
1970s	Schooling at leprosy camp and Ecole St Martin, Ayos.
1985	Trained as nursing aid (*aide soignant)* in Mbalmayo.
1986	Appointed to the rural health centre of Mefomo, Mefou.
1995	Appointment as nursing aid in Ayos Hospital.
2000–2002	Student at Ayos Nursing School and head nurse of Operating Theatre.
2002	Began working with Buruli ulcer projects in Ayos. The first Buruli health workers included: Mme Mbarga, Mr Same Ntanga, and ZB.
2006	Travelled to Switzerland for training at Centre hospitalier universitaire vaudois (CHUV) Lausanne. Training in Bénin.
2014	Retired from Ayos Hospital.

Both authors share an intimate personal and/or family experience of mycobacteria-induced wounds in leprosy and BU. Daniel Ze Bekolo personally experienced BU, then known as *Atom*, when he was 15 years old, and had to interrupt his school studies for one year. In 1989, one of his sons also developed a BU lesion on the leg. ZB himself treated it surgically, in combination with antibiotic therapy, without the guidance (and apparently against the will) of the supervising doctor, who had no experience of the disease. During our workshop in Ayos, Ze Bekolo and his son (also a nurse) showed us their scars. It is hard to overstate the directness of their experience of BU.

His successor as the BU surgeon in Ayos hospital, nurse Kombang Ekodogo, was born in 1962 in Ayos. His parents were patients living in the so-called “*léproserie*” (leprosy camp) of Ayos, immediately neighbouring the hospital site. He grew up experiencing the stigma associated with leprosy patients and their relatives. His training and his social standing made him a natural caregiver and community leader for residents of the leprosy camp. For both KE and ZB, the personal experience of NTDs such as leprosy and BU is presented as the main reason for their choice of the nursing profession:

“To tell you the truth, it is Buruli ulcer that led me to medicine, if one can say that. I met Buruli not as a health worker but in my flesh, that’s all”

(OI.ZB).

“If I may add something a little personal to what I have said about my career. Having known suffering personally…I have known what suffering is, and this has motivated me to choose medicine, to be able to help men who suffer”

(OI.KE).

Although such accounts may partly be “biographical illusions” [43], i.e., *a posteriori* re-interpretation of contingent choices, both insisted that they chose to work in leprosy and BU for “matters of heart.” The familial experience of BU and leprosy by ZB and KE enjoin us to take such life histories seriously in and of themselves. Both mycobacterial infections are extremely demanding for familial caregivers for multiple reasons, including the smell and visual aspects of lesions, the direct and indirect costs of treatment and mutilations, and, more generally, the patterns of social exclusion surrounding the diseases [21]. The chronicity of the diseases also requires time-consuming and repeated interventions on lesions and a long-term and holistic involvement with the patients as regards the management of disabilities and social rehabilitation. For both KE and ZB, these dimensions of mycobacterial NTDs were not textbook knowledge but daily aspects of their life since childhood. In oral interviews, they insist that their own first-hand experiences of chronicity, disability, and of the most prosaic aspects of lesion care were essential to their nursing practice.

#### 4. Long-term memory and Ayos as a multi-layered site of science and medicine

Local expertise on BU and individual biographies of care bring to bear on current therapeutic and research practice a longer-term history of medical care and research in Ayos. Furthermore, the present-day standing of Ayos as an international BU research site manifests the presence of this longer history at several levels.

The first continuity is ecological. The Nyong River is associated with both HAT and BU epidemics. In the first half the 20th century, the Nyong River economy and ecosystem channelled the emergence of the HAT epidemic, enabling both intense human–fly contacts and the spread of the disease among river-workers and travellers. Deforestation of the riverbanks to destroy tsetse habitats has been repeatedly undertaken in the name of HAT control since the German period. As documented by Giles Vernick and colleagues, the deforestation of the riverbanks was almost complete by the 1980s, following rising pressure for agricultural land and drought-associated fires [23]. It is now considered by oral histories and epidemiological studies to be the main environmental factor in increased incidence of BU since the 1980s. The radical change in the landscape of the Nyong River is thus both the consequence of public health interventions and the sign of an epidemiological transition from one NTD (HAT, now virtually absent from the region) to another (BU).

**Fig 3:**
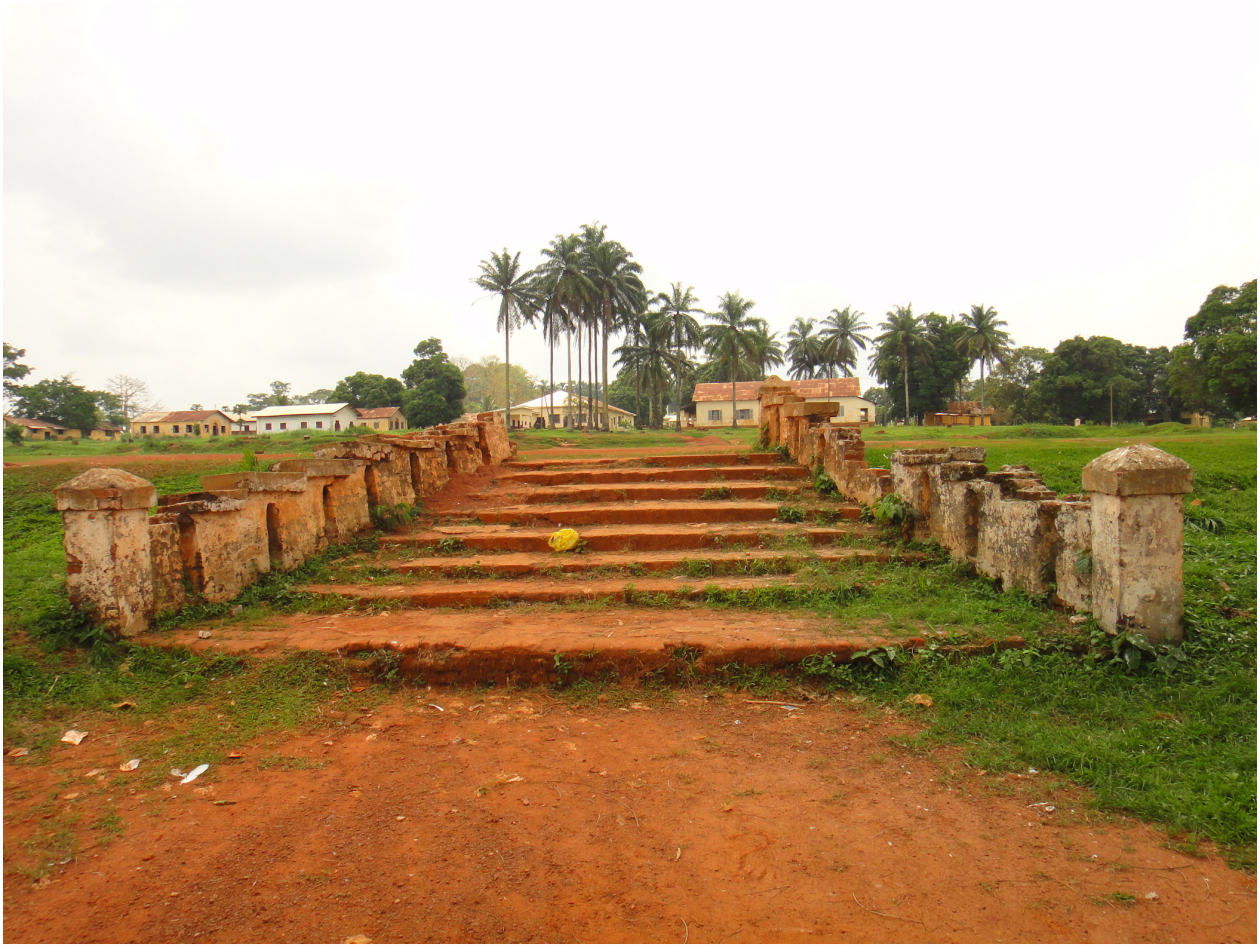
Remains of stairs inherited from the former sleeping sickness camp of Ayos. Image credit: John Manton, 2012.

The second continuity relates to the infrastructure inherited from HAT control (Fig 3), which extended its reach to other diseases, including leprosy and psychiatric disorders. The segregatory function of the site made Ayos a place of relegation, exclusion, and death. The epidemic of BU added a layer to that history of segregations: BU patients were treated at the leprosy camp until the 2000s, when a group of old wards inherited from the sleeping sickness camp were renovated and specifically reserved for BU patients. Concerns are often expressed that the concentration of BU patients in wards distinct from the main hospital may lead to the de facto creation of a “*Buruliserie*” analogous to past *léproserie* and *hypnoserie*, reiterating a history of stigmatization.

The connections of the leprosy camp to the humanitarian networks of leprosy relief, including the *Fondation Raoul Follereau* and the organization *Aide aux Lépreux Emmaus Suisse* (ALES), left a strong imprint on the response to BU, with ALES (now Fairmed) becoming the key actor and funder of BU research and care in Ayos. The infrastructure and expertise associated with the history of HAT and leprosy are thus built into current responses to BU.

Similar continuities mark local narratives about health workers. Witchcraft accusations about health workers inflicting the disease, or of benefiting from the extraction of body substances during treatment or surgery, echo similar accusations reported in the Ayos region during the sleeping sickness campaigns of the German and French period (OI.A8, [44]). More positively, our sources insisted on the ancient roots of the reputation of Ayos nurses in surgery. A French colonial doctor who worked in Ayos in the late 1940s told us that “the old Cameroonian nurses” present at the hospital at the time “taught him everything in surgery” (OI.P2). “Surgeons” (the title being given to nurses in the Ayos context) like Kombang are spontaneously situated in genealogies going back to the times of Dr Jamot and his *Jamotains* auxiliaries, who are considered the “founding fathers” of the hospital and town (OI.A9, OI.A10, OI.A14). For example, the surgeon of the hospital in the 1960s–1970s, Amba Mbida Benjamin (born in 1934), is himself the son of the *Jamotain* nurse Thomas Mbida Abada (1909–1971). He collaborated with Prof. Edzoa in the late 1970s and had Ze Bekolo as a student, who in turn trained Bitoto and Kombang (all of them being native to Ayos). Dense local social networks were thus involved in the transmission of surgical techniques, clinical knowledge, and expertise about BU. The recognition of this expertise is not merely an issue of equity but of intellectual and technical merit.

## Discussion: Care and Memory as Research Competencies

Providing more than merely historical perspective on BU in the Nyong area, this research argues that auxiliary health workers have developed specific forms of tacit, embodied, and expert knowledge that are crucial to clinical and epidemiological research on BU. We demonstrate that this knowledge is the product of multi-layered histories of medical care in the area, sedimented over a century of colonial and post-colonial medical activities. We propose taking seriously the concerns of auxiliary health workers about authorship and experimenting with participatory writing to recognize their contribution to our understanding of NTDs.

The local expertise of intermediaries is both a necessary and silenced dimension of the making of scientific knowledge. As scholarship in the history and sociology of science has shown, such tension between the local and the global is intrinsic to modern science [45]. On the one hand, science depends on localized arrangements, including divisions of labour across hierarchies of class, profession, race, and gender; on the other hand, scientific knowledge, to achieve global validity, has to be de-contextualized through material and literary operations [46]. In other words, the mediated nature of scientific facts is both fundamental and actively hidden.

In colonial contexts, Western scientific activities depended heavily on “middle figures,” such as translators and guides who acted as “knowledge brokers” and whose contributions were silenced by official histories [47]. In recent years, a new generation of historians have demonstrated that the “go-betweens” operating in the peripheries of European colonial empires have been fundamental actors in the emergence of modern science since the late 17th century. Our research participates in this reconsideration of auxiliaries as key mediators of science and medicine. The history of nurses in Ayos illustrates how, in the African context, subaltern staff trained as part of colonial medical campaigns had a long-lasting influence on medical training, scientific research, rural development, and nation building [44,48–52].

Our research tries to reverse the operation by which the tacit knowledge involved in research work is rendered invisible—involuntarily so, for the non-inclusion of subaltern staff in authors’ lists is standard practice and literally “goes without saying.” By focusing on the life histories of two nurses, we showed that their involvement in research was based on a personal and intimate experience of NTDs, and that their practice is anchored in historical and local trends in emerging infection and disease control. Their contribution not only consisted of their knowledge of the field, but more importantly of a specific competency acquired as care-receivers and caregivers. Our conclusions suggest, in line with the recent philosophical interest in the notion of care, that this task, generally performed by subaltern workers, is obscured by the hierarchies and norms of medical science [53]. The field of NTDs can be at the vanguard in questioning such hierarchies (especially those that are considered “natural”) and in making care and caregivers more visible as contributors and, indeed, authors, of scientific progress.

Our research finally calls for an increasing attention to history and memory in NTD research. Historical knowledge is more than simply perspectival; it can enrich the contexts and range of epidemiological research. As Tamara Giles Vernick and colleagues [23] recently showed, local narratives about the emergence of BU in the Nyong area highlighted key issues of environmental change and socio-economic crisis, which were hitherto blind spots of epidemiological studies, or which were only addressed through inappropriate discussions of “culture” and “beliefs” [22]. In this work, we showed that the graft of BU international research in Ayos in the 2000s occurred in an area where the expertise on BU was rooted in a century-long social history. This paper argues that the NTD research community must engage with this history and its actors.
